# Carboxyl-Terminal Modulator Protein Positively Acts as an Oncogenic Driver in Head and Neck Squamous Cell Carcinoma via Regulating Akt phosphorylation

**DOI:** 10.1038/srep28503

**Published:** 2016-06-22

**Authors:** Jae Won Chang, Seung-Nam Jung, Ju-Hee Kim, Geun-Ae Shim, Hee Sung Park, Lihua Liu, Jin Man Kim, Jongsun Park, Bon Seok Koo

**Affiliations:** 1 Department of Otolaryngology-Head and Neck Surgery, Research Institute for Medical Science, Chungnam National University, Daejeon, Republic of Korea; 2 Department of Medical Science, College of Medicine, Chungnam National University, Daejeon, Republic of Korea; 3 Research Institute for Medical Sciences and Pathology, College of Medicine, Chungnam National University, Daejeon, Republic of Korea; 4 Department of Pharmacology, Metabolic Diseases and Cell Signaling Laboratory, Research Institute for Medical Sciences, College of Medicine, Chungnam National University, Daejeon, Republic of Korea

## Abstract

The exact regulatory mechanisms of carboxyl-terminal modulator protein (CTMP) and its downstream pathways in cancer have been controversial and are not completely understood. Here, we report a new mechanism of regulation of Akt serine/threonine kinase, one of the most important dysregulated signals in head and neck squamous cell carcinoma (HNSCC) by the CTMP pathway and its clinical implications. We find that HNSCC tumor tissues and cell lines had relatively high levels of CTMP expression. Clinical data indicate that CTMP expression was significantly associated with positive lymph node metastasis (OR = 3.8, *P* = 0.033) and correlated with poor prognosis in patients with HNSCC. CTMP was also positively correlated with Akt/GSK-3*β* phosphorylation, Snail up-regulation and E-cadherin down-regulation, which lead to increased proliferation and epithelial-to-mesenchymal transition, suggesting that CTMP expression results in enhanced tumorigenic and metastatic properties of HNSCC cells. Moreover, CTMP suppression restores sensitivity to cisplatin chemotherapy. Intriguingly, all the molecular responses to CTMP regulation are identical regardless of p53 status in HNSCC cells. We conclude that CTMP promotes Akt phosphorylation and functions as an oncogenic driver and prognostic marker in HNSCC irrespective of p53.

Head and neck squamous cell carcinomas (HNSCCs) are the sixth most common cancer worldwide in men and occur as a heterogeneous tumor with an aggressive phenotype^1^. Despite the advances in biology and medicine over the past several decades, HNSCC remains a major cause of morbidity and mortality due to its relatively poor prognosis. Even with current treatment strategies, more than 50% of patients die from HNSCC or related conditions within 5 years^2^. This is most likely due to a lack of understanding about the molecular basis of HNSCC, and a lack of biomarkers that predict HNSCC progression or therapeutic resistance^3^. However, the development of HNSCC is characterized by multistep carcinogenic processes in which the activation of oncogenes and inactivation of tumor suppressor genes, including p53, epidermal growth factor receptor, Ras, MYC, survivin, cyclin D1, and cyclin-dependent kinase inhibitor, occurs as a result of genetic and epigenetic alterations. These alterations result in the proliferation and aggressiveness of tumor cells^4^.

Epithelial-to-mesenchymal transition (EMT) is a complex cellular process that is intimately linked to aggressiveness of cancer cells such as metastasis or resistance to chemotherapy^5^. Therefore, understanding EMT biology is essential to improve patient outcome. Previously, it is reported that both invasion and metastasis may be critically dependent on the acquisition by the incipient cancer cell of EMT features^6^. More recently, primary HNSCC tumors expressing a hallmark of EMT has a twofold increase in the metastasis compared to primary tumors without an EMT signature^7^,^8^. Despite the extensive research reported on signaling networks responsible for EMT, much remains to be understood regarding this dynamic cellular process^8^.

Recently, carboxyl-terminal modulator protein (CTMP) was shown to bind to the carboxy terminus of Akt and regulate its activity, although the role of CTMP in Akt regulation remains controversial^9^,^10^,^11^. Given that Akt signaling plays important roles in tumorigenesis and metastatic progression, by regulating apoptosis, as well as in cell cycling, protein synthesis, and glucose metabolism, understanding the role of CTMP in HNSCC may lead to new therapeutic targets. In addition, although cisplatin is the most used chemotherapy agent for HNSCC, only 30~40% of patients who had induction chemotherapy with cisplatin, achieved complete response, and there were still nearly 70~80% of patients treated for relapse or recurrent HNSCC showing no response^12^,^13^. Since PI3K/Akt activation is correlated with cisplatin resistance in HNSCC^14^, determining the relationship between CTMP and Akt regulation may contribute to our understanding of HNSCC chemoresistance. However, to the best of our knowledge, there are no studies about the role of CTMP in HNSCC.

In this study, we addressed CTMP expression and its role in Akt signaling during HNSCC development and progression were investigated using an *in vitro* functional assays and tissue microarray (TMA) expression analysis in different HNSCC patient cohorts. Furthermore, we aimed to determine whether CTMP expression could serve as a prognostic marker for tumor response to platinum-based chemotherapy.

## Materials and Methods

### HNSCC patients

We retrospectively reviewed the medical charts of 119 HNSCC patients who had undergone curative surgery (primary resection and appropriate cervical lymph node (LN) dissection according to disease stage) at the Department of Otolaryngology-Head and Neck Surgery of Chungnam National University Hospital from April 1999 to December 2011. This study was approved by the Institutional Review Board of Chungnam National University College of Medicine (Jung-gu Daejeon, Korea), and the informed consent requirement was waived. All experiments relating human tissue were performed in accordance with our institutional guidelines. Clinicopathological patient characteristics are summarized in Table 1. Of the patients, 40 (33.6%) had oral cavity cancer, 20 (16.8%) had oropharyngeal cancer, 11 (9.2%) had hypopharynx cancer, and 48 (40.4%) had larynx cancer. Tumor size and stage were classified according to the TNM system published by the American Joint Committee on Cancer (AJCC), and tumor differentiation was classified according to the World Health Organization (WHO) classification system. The mean follow-up duration was 40.6 months (range: 2–144 months).

### Tissue microarray (TMA) construction

Formalin-fixed paraffin-embedded tumor blocks were collected and TMAs were generated as described previously^15^. Briefly, sections were cut from each donor block and stained with hematoxylin and eosin (H&E) to identify the tumor area. A small tissue core (two cylinders per patient) with a diameter of 2 mm was taken from the donor block using a tissue chip microarray (Beecher Instruments, Silver Spring, MD, USA) and transferred to a recipient paraffin block. Histologic sections (5 μm thickness) were cut from the recipient paraffin block using standard techniques.

### Immunohistochemistry and scoring system

Immunohistochemical (IHC) staining using an anti-CTMP antibody (#4612, 1:50; Cell Signaling Technology Inc., Danvers, MA, USA) was performed using a 3,3′-diaminobenzidine (DAB) peroxidase substrate kit according to the manufacturer’s instructions (Sigma-Aldrich, St. Louis, MO, USA). At least two experienced pathologists analyzed slides under light microscopy (100x magnification) without accessing clinical patient information. Results were classified according to two parameters from a modified method described previously: CTMP staining extent (score: 0, no staining; 1, <35% positive cells; 2, 35–75%; 3, >75%) and staining intensity (score: 0, no staining; 1, weak; 2, moderate; 3, strong). By multiplying the staining extent by the intensity, we obtained the IHC staining grade (range: 0–9). For semi-quantitative analysis, grade 0 was considered no staining, grades 1 and 2 were considered weak staining (+1), grades 3 and 4 were considered moderate staining (+2), and grades 6 and 9 were considered strong staining (+3). Finally, for statistical comparison, specimens with no staining and (+1) were included in the low CTMP group, and those with (+2) and (+3) were included in the high CTMP group.

### Cell lines and reagents

The human HNSCC cell lines FaDu (hypopharynx), SNU1041 (hypopharynx), and SNU-1076 (larynx) were obtained from the Korean Cell Line Bank (Seoul, South Korea). Another HNSCC cell line, SCC15 (oral tongue), was kindly donated by Prof. C.H. Kim (Ajou University, Suwon, South Korea). Primary cultured human fibroblasts, kindly donated by Prof. J.H. Lee (Chungnam National University, Daejeon, South Korea), and HaCaT cells, derived from human keratinocytes and obtained from the American Type Culture Collection (Manassas, VA, USA) were used as normal epithelial cells. FaDu, SNU1041, and SNU1076 cells were maintained in RPMI 1640 medium (Gibco, Grand Island, NY, USA), while SCC15 cells were maintained in Dulbecco’s modified Eagle’s medium/Ham’s nutrient mixture F-12 (DMEM/F12; Gibco). HaCaT cells were maintained in high-glucose DMEM (Gibco). All cells were supplemented with 10% fetal bovine serum (FBS) and 100 U/mL penicillin-streptomycin (Gibco) and grown at 37 °C with 5% CO_2_ under humidified conditions. LY294002, an inhibitor of PI3 kinase-dependent Akt phosphorylation and kinase activity, was purchased from Cell Signaling Technology Inc. (#9901). Cisplatin was purchased from Sigma-Aldrich.

### RNA isolation and RT-PCR

Total cellular RNA was extracted using Trizol reagent (Invitrogen, Carlsbad, CA, USA), reverse transcribed, and amplified using specific primers for CTMP and glyceraldehyde 3-phosphate dehydrogenase (GAPDH), as described previously^16^. Primer sequences were: CTMP-F:5′-CTG GAA AGG TTT GCC TTC AT-3′/CTMP-R:5′-AGG TAA GGG CCT CCT TGA AA-3′; and PCR products were separated by electrophoresis on a 1% agarose gel containing ethidium bromide.

### Transient transfection of *carboxy-terminal modulator protein* (CTMP) cDNA and CTMP small interfering RNA (siRNA)

Transient transfection was performed once cells reached 60% confluence using Lipofectamine 2000 reagent (Invitrogen) following the manufacturer’s standard protocol. The full-length cDNA of human CTMP was amplified using PCR from cDNA libraries and inserted into the pCMV6-Entry (cat #PS100001) vector purchased from OriGene (Rockville, MD, USA). siRNAs for the control and CTMP (sense: 5′-CAC AUG GCA UUC CCU CUG U-3′; antisense: 5′-ACA GAG GGA AUG CCA UGU G-3′) were acquired from Bioneer (Daejeon, Korea).

### Cell proliferation assay

After transfection with control or CTMP cDNA or siRNA, cells were plated at a density of 5 × 10^3^/well in serum-free culture medium. After 48 h incubation for SNU1041 cells and 72 h for SCC15 cells, viability was measured using the Cell Proliferation Reagent WST-1 (Roche Diagnostics Corporation, Indianapolis, IN, USA) as described previously^7^,^17^. Results are presented as percentages relative to control cells.

### Cell invasion (transwell) assay

Transwell chambers (24-well, Costar, Cambridge, MA, USA) were used to assess cellular invasion as described previously^7^. Briefly, Matrigel (Coring, MA, USA) was dissolved in 100 μL MEM and applied to a polyethylene membrane filter with a pore size of 8 μm. Wells were coated overnight in a laminar flow hood. Next, cDNA or siRNA-transfected cells (1 × 10^4^ in 100 μL growth medium) were added to the top of the filter in the upper well. The chamber was incubated for 48 h for SNU1041 cells and for 72 h for SCC15 cells in 5% CO_2_ at 37 °C. Finally, attached cells in the lower section (invading cells) were stained with crystal violet and counted in four representative fields by light microscopy (200x magnification).

### Western blot analysis

Cells were lysed in lysis buffer containing 150 mM NaCl, 1.0% nonidet-P 40 (NP40), 0.5% sodium deoxycholate, 0.1% sodium dodecyl sulfate (SDS), 50 mM Tris, pH 8.0, and a protease inhibitor cocktail (Roche Applied Science, Vienna, Austria, pH 7.4). Electrophoresis was performed as described previously^7^.

The following primary antibodies were used for Western blot analysis: anti-CTMP, anti-vimentin, anti-Slug, anti-β-actin (1:1,000; Cell Signaling Technology Inc.), anti-E-cadherin, anti-Snail (1:1,000; Santa Cruz Biotechnology, Santa Cruz, CA, USA), anti-total Akt, -phopho-Akt (Ser 473), anti-total GSK3*β*, and -phospho-GSK3*β* (Ser 9) (Cell Signaling Technology). Following incubation with the corresponding horseradish peroxidase-conjugated secondary antibodies, immunoreactive bands were visualized by enhanced chemiluminescence (ECL) detection.

### Co-immunoprecipitation

To identify the interaction between Akt and CTMP, co-immunoprecipitation was performed using a mouse monoclonal antibody to Akt (Cell Signaling Technology). For immunoprecipitation, 200 μg of cell extract was precleared overnight at 4 °C with 10 μL of protein G-Sepharose beads pre-blocked with phosphate-buffered saline containing 20 mg/mL bovine serum albumin. One microgram of antibody was then added and samples were incubated at 4 °C for 2 h, followed by a 2 h incubation with 10 μL of pre-blocked protein G-Sepharose beads on a rotator. Beads were washed five times in lysis buffer. Samples were resolved by SDS-PAGE and detected by ECL.

### Statistical analysis

All statistical analyses were performed using SPSS for Windows statistical software (ver. 20.0; SPSS Inc., Chicago, IL, USA). Pearson’s chi-squared or Fisher’s exact tests were used to determine the relationship between CTMP expression and clinicopathologic parameters. Factors deemed of potential importance by univariate analyses were included in a multinominal logistic regression analysis to adjust for other factors. Survival curves were constructed using the Kaplan-Meier method, and a log-rank test was used to determine statistical significance.

All *in vitro* experiments were repeated three times, and statistical significance was analyzed using two-sided Student’s *t*-test or one-way analysis of variance (ANOVA) followed by Tukey’s post hoc test. Data are presented as means ± standard deviation (SD), and a *P* value <0.05 was considered statistically significant (*P < 0.05; **P < 0.01; ***P < 0.001).

## Results

### CTMP expression in HNSCC patient tissue

**Figure 1:**
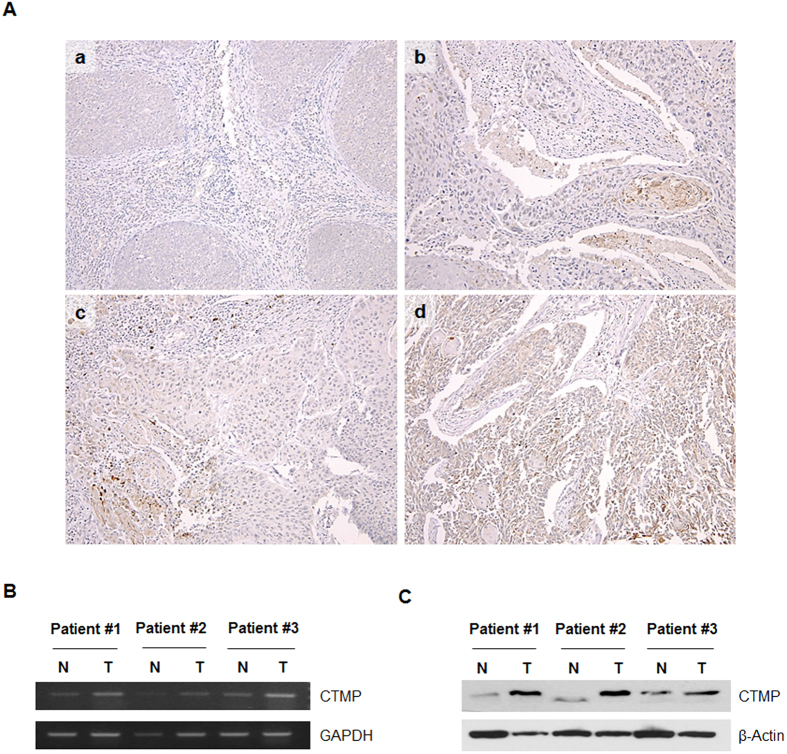
Carboxyl-terminal modulator protein (CTMP) expression in head and neck squamous cell carcinoma (HNSCC) clinical specimens. (**A**) Immunohistochemical CTMP expression. Representative immunohistochemical images of: (a) no staining intensity, (b) weak staining intensity, (c) moderate staining intensity, and (d) strong staining intensity in tumor samples from 119 HNSCC patients. (a) and (b) were classified as ‘low CTMP expression’, whereas (c) and (d) were classified as ‘high CTMP expression’ (100x magnification). (**B**,**C**) CTMP expression levels in the total cell lysate acquired from normal (N)–tumor (T) paired clinical specimens were examined through (**B**) RT-PCR and (**C**) Western blot assays.

To determine the clinical relevance of CTMP expression in human HNSCC, IHC staining of CTMP was performed on 119 paraffin-embedded samples harvested from HNSCC patients. Representative IHC images are shown in Fig. 1A. CTMP staining was detected in 68 of 119 (57.1%) HNSCC specimens, but was variably expressed. Eighty-six cases (72.3%; Fig. 1A-a,A-b) had low CTMP staining (0 and +1), 51 cases had no staining, and 33 cases (27.7%; Fig. 1A-c,A-d) had high staining (+2 and +3).

In addition, we assessed whether CTMP expression varied between normal and tumor tissue derived from the same patients at mRNA and protein levels. RT-PCR and Western blot analysis revealed that tumor tissues had higher CTMP expressions at both mRNA and protein levels compared to normal tissues (Fig. 1B,C). Therefore, we can assume that CTMP can be related with carcinogenesis in HNSCC.

### CTMP expression independently correlates with lymph node metastasis in HNSCC patients

We next evaluated the relationship between CTMP expression and clinicopathological characteristics. Of the various parameters described in Table 2, T classification, lymph node metastasis, and AJCC stage were significantly associated with CTMP expression. The three characteristics mentioned above were then modeled together using multinomial logistic regression to adjust for other factors. As shown in Table 3, tumors with high CTMP expression significantly increased the odds of lymph node metastasis by more than three-fold (odds ratio [OR] = 3.788, 95% confidence interval [CI] = 1.113–12.896, *P* = 0.033).

### CTMP expression is associated with poor prognosis in HNSCC

**Figure 2:**
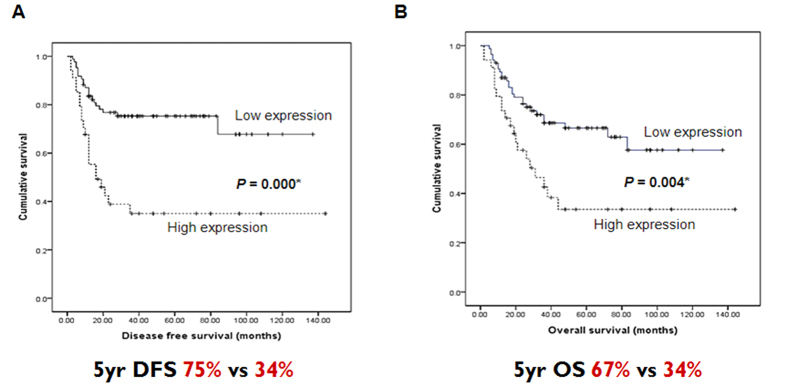
Kaplan-Meier plots for (**A**) disease-free survival (DFS) and (**B**) overall survival (OS) based on CTMP protein expression level as determined by immunoblotting in tumor tissues.

To examine the potential correlation between high CTMP expression and HNSCC survival rate, disease-free survival (DFS) and overall survival (OS) curves were calculated using the Kaplan-Meier method and compared using a log-rank test. As shown in Fig. 2A,B, patients with high CTMP expression had significantly lower DFS and OS rates than patients with low CTMP expression (*P* = 0.004 and P = 0.000, respectively). The 5-year DFS rate was 34.0% with high CTMP expression, but was 67% with low CTMP expression. The 5-year OS rate was 34.0% with high CTMP expression and 75.0% with low CTMP expression. Collectively, these data suggest that CTMP is a potential molecular biomarker for HNSCC aggressiveness and prognosis.

### CTMP expression in HNSCC cell lines

**Figure 3:**
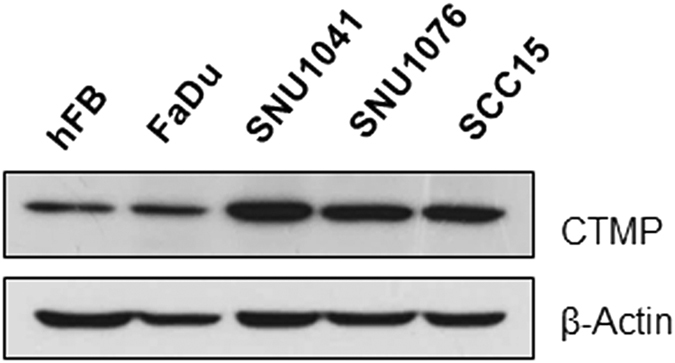
CTMP expression in HNSCC cell lines. Cell lysates were prepared from human fibroblast cells (hFB) and four HNSCC cell lines (FaDu, SNU1041, SNU1076, and SCC15) and examined by Western blot analysis using an anti-CTMP antibody. Representative images of three independent experiments are shown.

To confirm our observations from clinical data, we examined CTMP protein levels in a panel of HNSCC cell lines (FaDu, SNU1041, SNU1076, and SCC15) using normal fibroblasts (hFB) for comparison (Fig. 3). All HNSCC cells demonstrated relatively high CTMP expression compared to hFB, to a greater or lesser extent. Since CTMP was upregulated in tumors compared to normal tissues, we hypothesized that CTMP may be involved in HNSCC tumorigenesis. The SNU1041 and SCC15 cell lines were used for subsequent experiments because they derive from different head and neck areas (pharynx and oral cavity, respectively) and have different p53 genetic background, the most commonly mutated gene both in HNSCC tumors and cell lines^18^ (p53-wild-type^19^ or *-*mutated^20^, respectively).

### CTMP promotes proliferation of HNSCC cells

**Figure 4:**
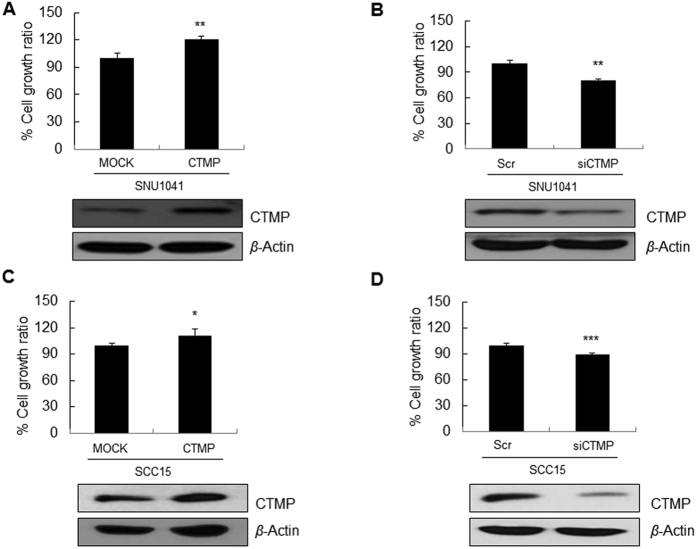
CTMP promotes HNSCC proliferation. To evaluate the effect of CTMP on cell proliferation, a WST-1 assay was performed after transient transfection of CTMP-specific cDNA (**A**,**C**) or small interfering RNA (siRNA) (**B**,**D**). CTMP protein levels after transfection were examined through Western blot analysis. Each figure is representative of three independent experiments. Scr, scrambled; *P < 0.05; **P < 0.01; ***P < 0.001.

The finding that T stage III and IV had a higher proportion of CTMP high than those of earlier stages led us to hypothesize that CTMP may drive cellular proliferation. To examine this hypothesis further, we performed a proliferation assay after CTMP up-/down-regulation in SNU1041 and SCC15 cell. As shown in Fig. 4, CTMP-overexpressing cells showed significantly increased proliferation (26.4%, *P* < 0.01; and 13.4%, *P* < 0.05, respectively, Fig. 4A,C) whereas CTMP-suppressed cells showed markedly decreased proliferation in both cell lines (20.5%, *P* < 0.01; and 11.7%, *P* < 0.001, respectively, Fig. 4B,D) suggesting contribution of CTMP signaling to cancer cell proliferation.

### CTMP positively regulates HNSCC cell invasiveness

**Figure 5:**
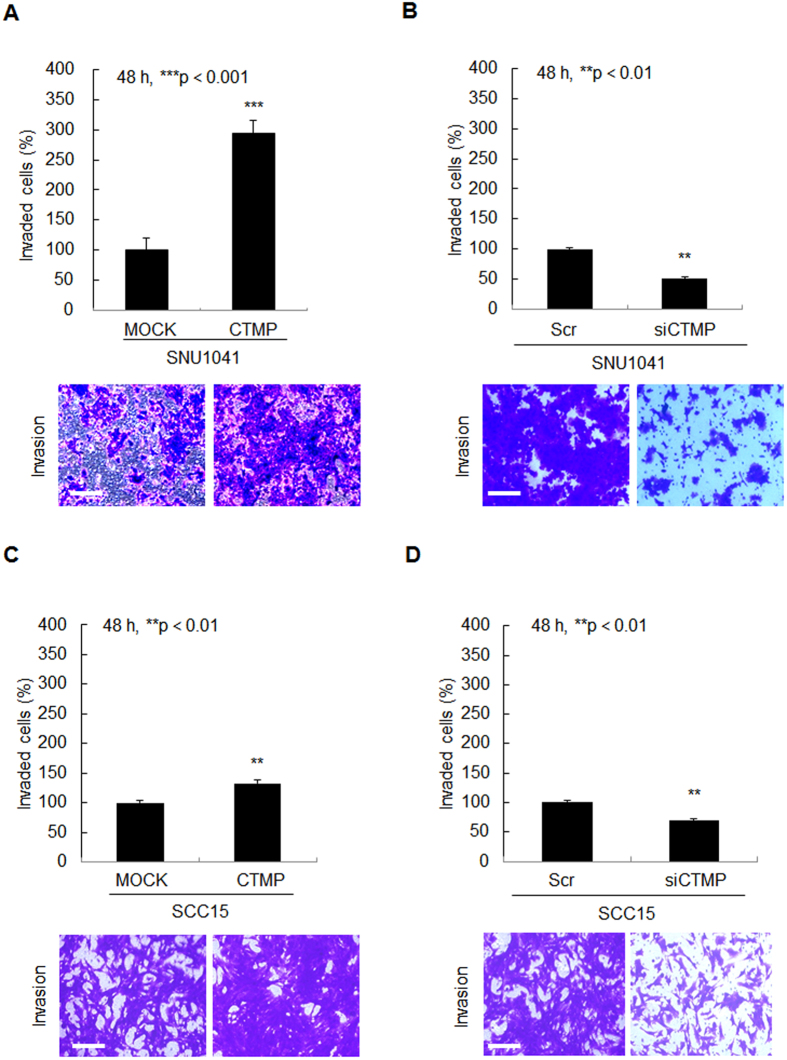
CTMP promotes HNSCC invasion. To elucidate the effect of CTMP on cell invasive phenotype, a Transwell chamber assay using Matrigel was performed. Each cell line was seeded onto a filter (pore size, 8 μm) coated with Matrigel in the upper chamber. After 48 h of transfection with CTMP-specific cDNA (**A**,**C**) or siRNA (**B**,**D**), the cells attached to the lower chamber were stained with crystal violet. Each figure is representative of three independent experiments. Scr, scrambled; **P < 0.01; ***P < 0.001. Scale bar = 50 μm.

Since CTMP-high tumors almost always presented with lymph node metastases (Table 1), we speculated that CTMP may promote invasive properties of HNSCC cells, which helps tumor cells to travel to a lymph nodes or distant site and to settle there. To test this *in vitro*, we performed a Transwell invasion assay using Matrigel, Invasion of cancer cells requires degradation of the basement membrane and extracellular matrix, cytoplasmic extension, and cell migration. Transwell invasion assay imitate this environment for tumor invasion. The attached cells in the lower section passed through the filter of the chamber, indicating invasive cells. As shown in Fig. 5, the number of cells that moved to the bottom chamber was significantly increased in CTMP-overexpressing SNU1041 and SCC15 cells (193.3%, *P* < 0.001; and 23.0%, *P* < 0.01, respectively, Fig. 5A,C), but markedly decreased in CTMP-suppressed cells compared to control cells (48.7%, *P* < 0.01 and 31.0%, *P* < 0.01, respectively, Fig. 5B,D) suggesting contribution of CTMP signaling to cancer cell migration and invasion.

### CTMP promotes epithelial-to-mesenchymal transition (EMT), associated with an increase in Snail

**Figure 6:**
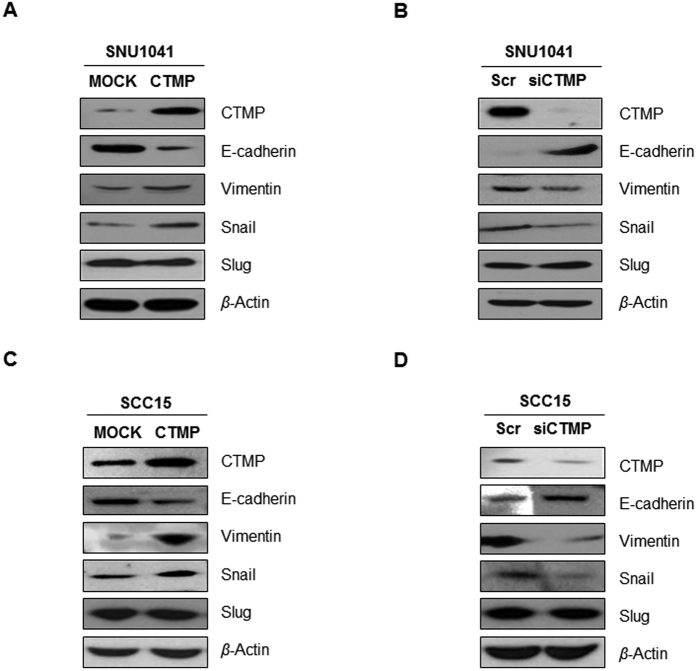
CTMP regulates E-cadherin and vimentin expression in a Snail-dependent/Slug-independent manner. Each cell line was transiently transfected with CTMP-specific cDNA (**A**,**C**) or siRNA (**B**,**D**). Expression of CTMP, E-cadherin (epithelial marker), epithelial-to-mesenchymal transition (EMT) markers (vimentin), and the level of EMT-inducing transcription factors (Snail and Slug) were measured by Western blot analysis. Each band is representative of three independent experiments. Scr, scrambled.

Within a primary tumor, a subpopulation of cells can undergo EMT, which confers novel migratory, invasive, and stem-like properties that promote metastasis. To examine whether CTMP promotes EMT, we examined both epithelial and mesenchymal markers and the well-known EMT-inducing transcription factors, Snail and Slug, by immunoblot analysis. As shown in Fig. 6A,C, CTMP-overexpressing cells exhibited a significant decrease in E-cadherin, while the mesenchymal markers vimentin and Snail were increased in both SNU1041 and SCC15 cells. In addition, CTMP-silenced cells showed the exact opposite tendency; up-regulation of E-cadherin and down-regulation of vimentin and Snail (Fig. 6B,D). However, there were no significant changes in the expression of Slug (Fig. 6). These data mean that CTMP promotes Snail-associated EMT.

### CTMP binds Akt in HNSCC cells and upregulates Akt signaling

**Figure 7:**
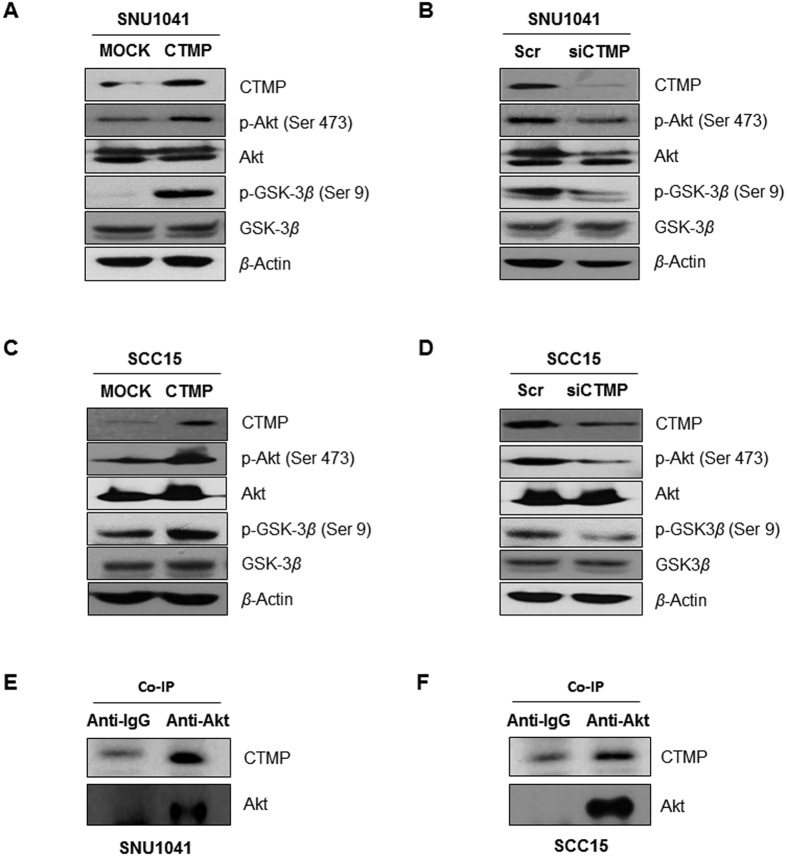
CTMP regulates PI3K-mediated Akt phosphorylation through an epithelial growth factor receptor (EGFR)-independent pathway. (**A**–**D**) Each cell line was transiently transfected with CTMP-specific cDNA (**A**,**C**) or siRNA (**B**,**D**) and the expression of Akt, p-Akt at Ser 473, GSK-3*β*, and p-GSK-3*β* at Ser 9 were analyzed through Western blot analysis. (**E**,**F**) The cell lysates from each cell line were co-immunoprecipitated with an anti-Akt antibody, and the immunoprecipitation product was analyzed through Western blot for CTMP and Akt. (**G**) To determine the sequential relationship between Akt and CTMP, the expression of Akt, p-Akt at Ser 473, and CTMP were examined after LY294002 (15 μm for 24 h) treatment by Western blotting. (**H**) To elucidate the relationship between EGFR and CTMP signal pathways, the expression of Akt, p-Akt at Ser 473, and CTMP were examined after EGF and/or LY294002 treatment by Western blotting. EGF treatment additively promoted Akt phosphorylation compared to controls without altering CTMP expression.

Akt signaling is one of the most important oncogenic driver in a wide range of human cancers including HNSCC^9^,^21^, thus understanding the regulatory mechanism of Akt is important for developing therapeutic tactics against cancers. Although, CTMP has been emerged as one of Akt regulating protein, there are disparate views regarding the direction of regulation and no report about the association between CTMP and Akt regulation^10^,^11^. To evaluate the association between CTMP expression and Akt activation, we evaluated Akt phosphorylation at Ser 473 after CTMP up-/down-regulation. Although, Akt is activated via two sequencial phosphorylation (Thr 308 is followed by Ser 473), we examined only Ser 473 phosphorylation, because additional phosphorylation at Ser 473 is essential to accomplish Akt full activation in spite of Thr 308 phosphorylation^9^. As shown in Fig. 7A,C, CTMP up-regulation markedly enhanced Akt phosphorylation without changing total Akt amount. In contrast, CTMP down-regulation significantly attenuated Akt phosphorylation with stationary expression of total Akt (Fig. 7B,D). Moreover, the phosphorylation of GSK-3*β*, the downstream protein kinase of Akt, at Ser 9 was increased or decreased after CTMP-overexpression/-suppression, respectively, indicating that CTMP regulates Akt activity (Fig. 7A–D).

Furthermore, to determine the direct interaction between CTMP and Akt, co-immunoprecipitation was performed. The cell lysate of SNU1041 and SCC15 cell lines was precipitated using an anti-Akt antibody. CTMP was detected in the anti-Akt-IP products but CTMP was not detected in normal Ig G immunoprecipitates, indicating that there is a direct association between CTMP and Akt proteins (Fig. 7E,F). Taken together, CTMP binds Akt and upregulates Akt pathway in SNU1041 and SCC14 cells.

### CTMP suppression increases cisplatin cytotoxicity in HNSCC cells

**Figure 8:**
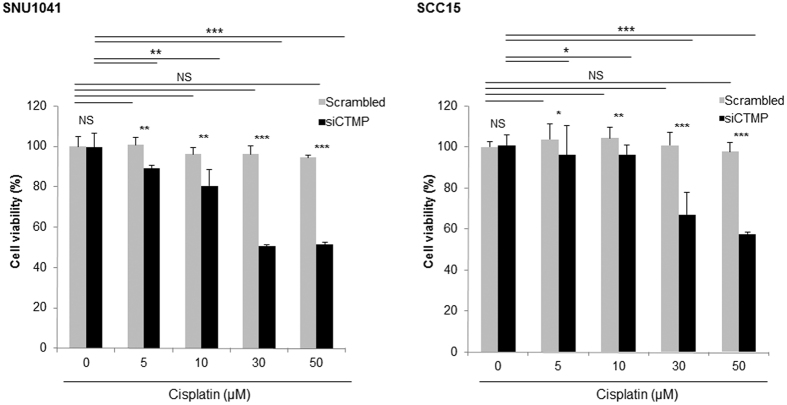
CTMP suppression increases cisplatin cytotoxicity in HNSCC cells. Following exposure of the indicated cells to various concentrations of cisplatin for 24 h, cell viability was measured using a WST-1 proliferation assay. Although the cell lines were resistant to cisplatin treatment, CTMP siRNA-transfected cells demonstrated marked cisplatin-induced cytotoxic effects compared with control siRNA-transfected cells. Data represent the means ± SD of three independent experiments. NS, not significant; *P < 0.05; **P < 0.01; ***P < 0.001.

Cells that undergo EMT often acquire properties associated with cancer stem cells, which are related to chemoresistance^22^. Therefore, given that CTMP is associated with EMT in HNSCC cells, we hypothesized that CTMP affects chemoresistance and aimed to determine the role of CTMP silencing on cisplatin efficacy in SNU1041 and SCC15 cells. As shown in Fig. 8, even 50 μM of cisplatin did not significantly attenuate cell viability in SNU1041 or SCC15 cell lines, both of which have high endogenous CTMP expression. However, although CTMP knockdown alone did not have significant effect, cisplatin decreased cell viability in CTMP-suppressed cells in a dose dependent manner, indicating that CTMP-suppression restores sensitivity to cisplatin treatment.

To sum up our *in vitro* data, CTMP acts as an oncogenic driver in HNSCC via promoting Snail-associated EMT and Akt phosphorylation. Moreover, CTMP suppression increased chemosensitivity.

## Discussion

In general, the transformation of normal epithelium to squamous cell carcinoma (SCC) occurs in multiple steps, involving the sequential activation of oncogenes and the inactivation of tumor suppressor genes^23^. Although remarkable progress has been made in the identification of altered tumor onco-/suppressor-genes and their related protein products in HNSCC^23^, the proliferative pathways driving uncontrolled cell growth are still poorly defined, which limits our ability to identify more comprehensive mechanism-based therapeutic approaches. In addition, given the minimal success of EGFR targeting strategy and the commonly occurring resistance^24^, the need for additional treatment options that improve outcome of existing therapeutic tactics for HNSCC is pressing.

Akt (also called protein kinase B) is a major downstream target of receptor tyrosine kinases that signal via phosphatidylinositol 3-kinase. Recently, dysregulation of Akt activity has been found in a wide range of human cancers including HNSCC, and the Akt signaling pathway has emerged as a key regulator of cell growth and decision of cell fate^25^,^26^. Of the genetic or epigenetic alterations known in HNSCC, one of the most frequently altered pathways is EGFR/Ras/PI3K, which, along with other pathways (p53/DNp63, pRb/CycD1, TGF-β/Smad and NF-κB), can lead to Akt dysregulation which has indeed been found in 20~60% of tumor samples and in the majority of HNSCC-derived cell lines^25^,^27^,^28^,^29^. Moreover, Akt overexpression is predictive of poor clinical outcome, and is associated with advanced disease, local recurrence, and decreased survival^30^,^31^,^32^. Given the importance of Akt in HNSCC, understanding the regulatory mechanisms of Akt is important to develop new therapeutic strategies against HNSCC.

Akt activation is managed by various interacting proteins and upstream regulators. Since Maira *et al*. published the original study describing 27-kDA CTMP as an Akt-interacting protein in 2001^11^, accumulating evidence suggests that CTMP binds to the Akt carboxy terminus and negatively regulates its activity in lung cancer^33^, glioblastoma^34^,^35^, pancreatic adenocarcinoma^36^, and cervical cancer cell lines^37^, suggesting CTMP is a tumor suppressor. However, opposite results were found by Ono *et al*.^38^. In addition, Liu *et al*. found that CTMP is a positive regulator of Akt phosphorylation and is an oncogenic driver in breast cancer^9^. These conflicting results could be due to different biological responses between different cell types. Therefore, determination of the role of CTMP in HNSCC is needed, and no previous studies have examined this. Our results show that CTMP functions as a positive regulator of Akt, and has oncogenic activity in HNSCC.

We also found that there is a biological link between CTMP and cellular invasion of HNSCC cells, which is essential in tumor progression and metastasis. Since HNSCC is characterized by local invasion and lymphatic metastasis, understanding the molecular mechanisms that mediate tumor invasion and metastasis is critical for the identification of novel therapeutic targets. To verify the effect of CTMP-Akt regulation on invasive cellular phenotypes at the molecular level, we analyzed protein levels of Slug and Snail, transcription factors and master regulators of EMT, and the levels of E-cadherin and vimentin, cellular machinery proteins associated with the invasion and thus hallmarks of EMT^8^,^39^. In our data, CTMP upregulation decreased E-cadherin expression and increased the expression of vimentin and Snail without altering Slug signaling, suggesting Snail-dependent/Slug-independent E-cadherin regulation and EMT induction in HNSCC cell lines. Although, further studies are needed to figure out potential association with other EMT activating transcription factors such as Twist and Zeb1/2, to the best of our knowledge, this is the first evidence that CTMP modulates EMT in cancer. In addition, consistent with the *in vitro* EMT results, we demonstrated that CTMP was upregulated in human HNSCC and was positively associated with LN metastasis and poor prognosis in HNSCC patients.

Recently, Akt has been shown to repress the transcription and protein expression of E-cadherin, resulting in EMT^40^. Grille *et al*. showed that SCC cells producing a constitutively active form of Akt produce a transcription factor, Snail, which represses the expression of the E-cadherin gene^40^. In addition, Akt induces the inactivation (phosphorylation) of GSK-3β, which suppresses the phosphorylation of Snail. This results in Snail nuclear localization and up-regulation (due to protein stabilization) and E-cadherin downregulation, which subsequently results in EMT^41^,^42^. The role of the Akt/GSK-3*β*/Snail pathway in EMT has been reported previously in hepatocellular carcinoma^43^ and in a gefitinib-resistant HNSCC cell line^44^. Consistent with the previous reports, we found that Akt/GSK-3*β* and Snail were simultaneously up/down regulated by CTMP, while E-cadherin expression had the opposite reaction. Thus, as a downstream of CTMP, the Akt/GSK-3*β*/Snail/E-cadherin pathway is likely involved in EMT in HNSCC cell lines. Additionally, in consistent with previous study showed that downregulation of E-cadherin enhances proliferation of HNSCC through transcriptional regulation of EGFR^45^, CTMP up-regulation, which significantly decreased E-cadherin expression in both SNU1041 and SCC15 cell lines, promoted cell proliferation and vice versa.

Intriguingly, we also found that both cell lines, despite harboring different p53 genetic backgrounds, demonstrated the same molecular response to CTMP up-/down-regulation in all experiments. Given that gene expression differences are related to heterogeneous biological responses such as sensitivity to chemotherapy and malignancy^46^, and that p53 mutations are involved in resistance to platinum based^47^ and EGFR-targeting therapies^48^, these findings suggest that CTMP targeting stratagem may possess general anticancer efficacy regardless of p53 status. Thus, we can highlight that further studies should be performed to determine the potential of CMTP targeting to overcome chemoresistance.

In conclusion, our data suggest that CTMP functions as a positive regulator of Akt and facilitates HNSCC invasion such as LN metastasis by regulating EMT in a Snail-dependent manner indicating the oncogenic activity of CTMP in HNSCC, and its expression is an independent predictor of clinical prognosis and response to platinum-based chemotherapy in HNSCC. Given the data in this study, examining CTMP further in patient tumor tissues could identify patients at risk for tumor progression and chemotherapy resistance. Moreover, if validated in further studies including *in vivo* and clinical trial, a CTMP-targeting agent partnered with an existing anticancer modality could be a novel tactic to overcome drug resistance and ultimately improve HNSCC outcomes.

## Additional Information

**How to cite this article**: Chang, J. W. *et al*. Carboxyl-Terminal Modulator Protein Positively Acts as an Oncogenic Driver in Head and Neck Squamous Cell Carcinoma via Regulating Akt phosphorylation. *Sci. Rep.* **6**, 28503; doi: 10.1038/srep28503 (2016).

## Figures and Tables

**Table 1:** Clinicopathological characteristics of 119 HNSCC patients.

**Characteristics**	**Total**	**Primary site (number)**			
		Oral cavity(N = 44)	Oropharynx(N = 17)	Hypopharynx(N = 10)	Larynx(N = 48)
Age					
Range (year)	25–87	25–87	42–71	46–72	49–83
Mean (year)	62	59	58	63	66
Gender					
Male	105	34	15	10	46
Female	14	10	2	0	2
Tumor differentiation					
Well	33	17	3	2	11
Moderate	56	21	10	6	19
Poor	30	6	4	2	18
Tumor size					
T1	40	26	5	2	7
T2	26	11	9	2	4
T3	19	3	2	1	13
T4	34	4	1	5	24
Nodal involvement					
N−	68	30	3	2	25
N+	51	14	14	8	23
AJCC Stage					
I	27	20	1	1	5
II	16	7	2	1	3
III	16	5	0	1	9
IV	60	112	14	7	31
PostOP RTx					
Yes	47	26	4	4	13
No	72	18	13	6	35

AJCC, American Joint Committee on Cancer; PreOP RTx, preoperative radiotherapy.

**Table 2:** Association between CTMP expression and various clinicopathological features of 119 HNSCC patients.

**Variables**	**No. of patients**	**CTMP expression**		
		**Low**	**High**	***P***
Age (year)				1.000
<65	63	45	18	
≥65	56	40	16	
Gender				1.000
Male	105	75	30	
Female	14	10	4	
T stage				0.024*
I + II	66	53	13	
III + IV	53	32	21	
LN metastasis				0.000*
No	60	54	6	
Yes	59	31	28	
AJCC stage				0.000*
I + II	41	39	2	
III + IV	78	46	32	
Histological grade				0.382
Well	33	26	7	
Moderate	56	40	16	
Poor	30	19	11	

CTMP, Carboxyl-terminal modulator protein; LN, lymph node; AJCC, American Joint Committee on Cancer. *P < 0.05 between the two categories for a given variable.

**Table 3:** Multinomial logistic regression for the association of CTMP expression with T classification, lymph node metastasis, and AJCC stage.

**Factor**	***β***	***P*****value**	**Exp (*****β***)	**95% CI**
Advanced T stage	0.200	0.703	1.221	(0.438, 3.405)
Positive LN metastasis	1.332	0.033*	3.788	(1.113, 12.896)
Advanced AJCC stage	1.500	0.136	4.483	(0.623, 32.268)

Exp (*β*) indicates odd ratio; CI, confidence interval; LN, lymph node; AJCC, American Joint Committee on Cancer. *P < 0.05 between the two categories for a given variable.
